# Humoral and Cellular Response to Spike of Delta SARS-CoV-2 Variant in Vaccinated Patients With Multiple Sclerosis

**DOI:** 10.3389/fneur.2022.881988

**Published:** 2022-05-31

**Authors:** Linda Petrone, Carla Tortorella, Alessandra Aiello, Chiara Farroni, Serena Ruggieri, Concetta Castilletti, Silvia Meschi, Gilda Cuzzi, Valentina Vanini, Fabrizio Palmieri, Luca Prosperini, Shalom Haggiag, Simona Galgani, Alba Grifoni, Alessandro Sette, Claudio Gasperini, Emanuele Nicastri, Delia Goletti

**Affiliations:** ^1^Translational Research Unit, National Institute for Infectious Diseases Lazzaro Spallanzani-IRCCS, Rome, Italy; ^2^Department of Neurosciences, San Camillo-Forlanini Hospital, Rome, Italy; ^3^Department of Human Neurosciences, Sapienza University of Rome, Rome, Italy; ^4^Neuroimmunology Unit, IRCSS Fondazione Santa Lucia, Rome, Italy; ^5^Laboratory of Virology, National Institute for Infectious Diseases Lazzaro Spallanzani-IRCCS, Rome, Italy; ^6^Unità Operativa Semplice (UOS) Professioni Sanitarie Tecniche, National Institute for Infectious Diseases Lazzaro Spallanzani-IRCCS, Rome, Italy; ^7^Respiratory Infectious Diseases Unit, National Institute for Infectious Diseases Lazzaro Spallanzani-IRCCS, Rome, Italy; ^8^Center for Infectious Disease and Vaccine Research, La Jolla Institute for Immunology (LJI), La Jolla, CA, United States; ^9^UOC Malattie Infettive ad Alta Intensità di Cura, National Institute for Infectious Diseases Lazzaro Spallanzani-IRCCS, Rome, Italy

**Keywords:** multiple sclerosis, COVID-19, vaccine, immune response, T-response, DMTs

## Abstract

**Objectives:**

We assessed vaccination-induced antibody and cellular response against spike from the ancestral strain and from the Delta Severe Acute Respiratory Syndrome CoronaVirus-2 (SARS-CoV-2) variant in patients with Multiple Sclerosis (MS) treated with disease modifying treatments.

**Methods:**

We enrolled 47 patients with MS and nine controls (“no MS”) having completed the vaccination schedule within 4–6 months from the first dose. The Interferon (IFN)-γ-response to spike peptides derived from the ancestral and the Delta SARS-CoV-2 was measured by enzyme-linked immunoassay (ELISA). Anti-Receptor Binding Domain (RBD) IgG were also evaluated.

**Results:**

No significant differences were found comparing the IFN-γ-specific immune response between MS and “no MS” subjects to the ancestral (*P* = 0.62) or Delta peptide pools (*P* = 0.68). Nevertheless, a reduced IFN-γ-specific response to the ancestral or to the Delta pools was observed in subjects taking fingolimod or cladribine compared to subjects treated with ocrelizumab or IFN-β. The antibody response was significantly reduced in patients with MS compared to “no MS” subjects (*P* = 0.0452) mainly in patients taking ocrelizumab or fingolimod.

**Conclusions:**

Cellular responses to Delta SARS-CoV-2 variant remain largely intact in patients with MS. However, the magnitude of these responses depends on the specific therapy.

## Introduction

Multiple sclerosis (MS) is an autoimmune disorder of the central nervous system (1, 2). The treatment of MS includes several drugs, called disease modifying treatments (DMTs) (1, 2). DMTs may affect humoral and cellular immunity (1), which are both essential for controlling Severe Acute Respiratory Syndrome CoronaVirus-2 (SARS-CoV-2) infection (3–5). Among patients with MS under DMTs, anti-CD20 monoclonal antibodies (mAb) treatment is associated with an increased risk for severe disease (6–8).

Disease modifying treatments may also impair vaccine-induced immune responses (9). Indeed, recently data demonstrated a lower humoral and cellular response in vaccinated patients with MS treated with fingolimod or ocrelizumab compared to vaccinated health-care workers (9, 10).

Furthermore, also viral variants should be taken into account as potential weakening factors for vaccine-induced protection in patients with MS. In particular, it would be useful to assess, if vaccine-induced cross-reactive immune responses may control infection and disease severity against viral variants. SARS-CoV-2 Delta variant was recognized responsible of an exponential increase of infections and deaths (11). In the last 2 months, Omicron variant is rapidly replacing all the other viral variants, including Delta (12). Indeed, compared to the Delta variant, Omicron infects cells of upper respiratory tract rather than the lungs, and this contributes to its rapid spreading. Moreover, infections by Omicron variant seems to cause lower clinical severity outcomes compared to Delta (12). The T-cell responses to both Omicron and Delta variant are detectable in mRNA-vaccinated patients with MS treated with anti-CD20; however, the magnitude of the response to Delta and Omicron was lower compared to that found to vaccine strain (13). However, so far no data are available on the cellular response to Delta variant across patients with MS treated with different DMTs.

In this study, we assessed the cellular response against Delta SARS-CoV-2 variant following COVID-19 vaccination in patients with MS treated with the following DMTs: anti-CD20 (i.e., ocrelizumab), sphingosine-1-phosphate receptor modulators (i.e., fingolimod), synthetic purine nucleoside (i.e., cladribine) and immunomodulators (i.e., Interferon-β). The humoral response concomitantly was evaluated.

## Materials and Methods

### Study Population

Patients with MS were enrolled at San Camillo Forlanini Hospital (Approval number 831/CE Lazio 1). COVID-19 vaccinated subjects with no history of MS (“no MS”) were included as controls and were enrolled at INMI (Approval numbers 297/2021; 319/2021; 72/2015 and 59/2020). This is a prospective study and subjects within both groups were enrolled based on the same time frame of the vaccination schedule, e.g., a vaccination schedule completed within 4 and 6 months from the first vaccine dose. Written informed consent was required to participate to the study. Patients with MS were part of a cohort previously described (9). MS diagnosis was based on McDonald 2017 Criteria (14). The therapy regimens considered were: ocrelizumab, fingolimod, cladribine, and IFN-β. The exclusion criteria for patients with MS were age <18 and >70 years, previous COVID-19 or present SARS-CoV-2 infection, previous treatment with corticosteroids in the last 30 days before enrollment, DMTs treatment duration <12 months, pregnancy or breastfeeding, liver of kidney failure, cancer, and immunodeficiency. The exclusion criteria for “no MS” subjects were age <18 and >80 years, immunosuppressive drugs, chronic diseases as diabetes and autoimmune disorders, HBV/HCV/HIV infection, pregnancy. Clinical and demographical info were recorded at enrollment.

### Stimuli

“Ancestral” and “Delta” spike SARS-CoV-2 peptide pools, designed and carried out on the Wuhan-Hu-1 strain (GenBank ID: MN908947) or on the GISAID ID: EPI_ISL_2020950, were used, respectively. Peptide pools consisted of overlapping 15-mers by spanning the entire spike proteins (*n* = 253). Peptide pools were generated based on spike composition and were used at 0.1 μg/ml.

Staphylococcal Enterotoxin B (SEB) antigen (sigma Aldrich) was used at 200 ng/ml.

### IFN-γ Whole-Blood Assay

Interferon-γ level was measured after whole blood stimulation with SARS-CoV-2 peptide pools as reported (15–17) and measured by an automatic ELISA (ELLA, protein simple). Whole blood left unstimulated or stimulated with SEB served as negative or positive controls, respectively.

### Serology

Anti-Nucleoprotein IgG (Anti-N IgG) and anti-Receptor Binding Domain (RBD) IgG (Architect® i2000sr Abbott Diagnostics, Chicago, IL) were evaluated as reported (9, 18). Anti-N-IgG index values ≥ 1.4 or anti-RBD-IgG binding antibody units (BAU)/ml ≥ 7.1 were considered positive.

### Statistics

The results were analyzed using GraphPad software (GraphPad Prism 8 XML ProjecT). Median and interquartile range (IQR) were calculated. Kruskal–Wallis test for comparisons among groups was used. Moreover, Mann–Withney *U* test as non-parametric test for unpaired data and the Wilcoxon matched-pairs signed rank test as nonarametric test for paired data were used. Bonferroni correction was applied when appropriate. Chi-square test was used for categorical variable. Correlations were calculated using Spearman's Rank test and *r*_s_ > 0.7 was considered high correlation, 0.7 < *r*_s_ > 0.5 moderate correlation and *r*_s_ < 0.5 low correlation. Differences were considered significant when *P* < 0.05 or *P* < deriving from Bonferroni correction.

## Results

### Characteristics of the Enrolled Subjects

We enrolled 47 patients with MS and nine patients with “no MS”. The two populations were similar for age, gender, origin distribution (Table 1) and lymphocyte counts (Table 1, Supplementary Figure 1A). Patients with MS were stratified according to DMTs in four groups: ocrelizumab (*n* = 10), fingolimod (*n* = 9), cladribine (*n* = 13), IFN-β (*n* = 15). The group of “no MS” subjects included five healthy donors (three of them were health care workers), three subjects with tuberculosis infection, and one subject with pneumonia (not COVID-19 related). All MS patients received mRNA vaccines, whereas, within “no MS” subjects, 6/9 received mRNA vaccines and 3/9 received viral vector-based vaccines (Table 1). Table 1 shows the demographical and clinical info of the enrolled subjects.

**Table 1:** Demographical and clinical characteristics of the enrolled subjects.

	**MS**	**No MS**	***P*-Value**
***N*** **(%)**	47	9 (17.3)	
**Age median (IQR)**	48 (37–54)	28 (25–53)	0.11^*^
**Female** ***N*** **(%)**	23 (48.9)	5 (55.6)	0.72^**^
**Origin** ***N*** **(%)**			
West Europe	46 (97.9)	9 (100.0)	0.66 ^**^
East Europe	0 (0)	0 (0)	
Africa	0 (0)	0 (0)	
Sud America	1 (2.1)	0 (0)	
**Positive anti-RBD IgG** ***N*** **(%)**	34 (72.3)	9 (100)	<0.0001^**^
**Positive anti-N IgG** ***N*** **(%)**	0 (0)	0 (0)	–
**Diagnosis of “no MS” subjects** ***N*** **(%)**			
Healthy donors	–	5 (55.6)	
Tuberculosis infection	–	3 (33.3)	
Pneumonia (not COVID-19 related)	–	1 (11.1)	
**Vaccine type** ***N*** **(%)**			
mRNA	47 (100.0)	6 (66.7)	<0.0001^**^
Viral vector	–	3 (33.3)	
**Lymphocyte counts ×10** ^**3**^ **/μl** ^**§**^ **median (IQR)**	1.3 (0.85–1.75)	1.6 (0.95–1.8)	0.61^*^
**Treatment** ***N*** **(%)**			
Ocrelizumab	10 (21.3)	–	
Fingolimod	9 (19.1)	–	
Cladribina	13 (27.7)	–	
IFN-β	15 (31.9)	–	

*N, number; IQR, interquartile range; RBD, receptor binding domain; IFN, interferon*.

§ *Data available in 44/47 MS patients and 4/9 “no MS” subjects*.

* *Mann–Whitney test*.

** *Chi-square test*.

### Similar IFN-γ-Specific Response to Spike From Delta SARS-CoV-2 Variant in MS Patients and Controls

**Figure 1:**
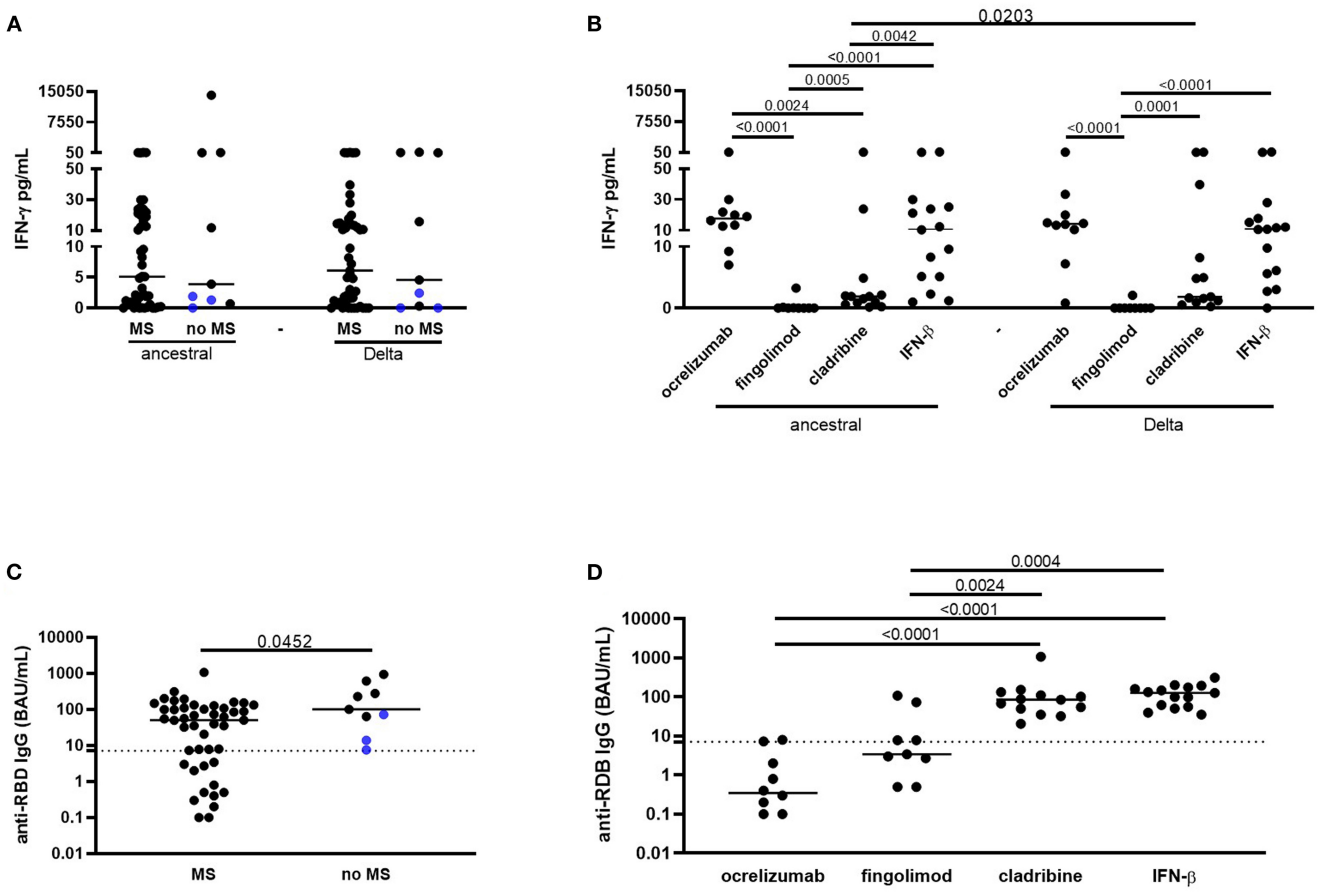
Vaccinated patients with MS show a T-cell-specific response to spike from Delta SARS-CoV-2 variant and an anti-RBD IgG response similar to control subjects, although DMTs differently affect the responses. **(A)** No significant differences were found comparing the IFN-γ level between MS patients and “no MS” subjects in response to the ancestral or to Delta spike. The IFN-γ response to the ancestral peptide pool was similar to that observed in response to the Delta peptide pool in both patients with MS and in “no MS” subjects. **(B)** Patients with MS treated with fingolimod and cladribine showed a significant lower IFN-γ-specific response to both the ancestral or to the Delta pools compared to subjects treated with ocrelizumab, or IFN-β. **(C)** Patients with MS showed significant lower anti-RBD-IgG levels compared to “no MS” subjects. **(D)** MS patients treated with ocrelizumab or fingolimod had significant lower anti-RBD-IgG levels compared to patients treated with cladribine or IFN-β. Horizontal lines represent medians. Dotted lines represent ELISA IgG cut-off. IFN-γ levels were measured by ELLA, anti-RBD IgG were measured by ELISA. Mann–Whitney or Wilcoxon tests were used for pairwise comparisons. MS, multiple sclerosis; IFN, interferon; RBD, receptor binding domain; BAU, binding antibody units.

No significant differences were found comparing the IFN-γ level between patients with MS and patient without MS in response to the ancestral (MS median 5.11 pg/ml, IQR: 0.87–19.9; no MS median 3.89 pg/ml, IQR: 1–79; *P* = 0.62) or to Delta peptide pool (MS median 6.1 pg/ml, IQR: 0.82–14.9; no MS median 4.56 pg/ml, IQR: 0.16–95.2; *P* = 0.68; Figure 1A). Moreover, the IFN-γ response to the ancestral peptide pool was similar to that observed in response to the Delta peptide pool in both patients with MS (ancestral: median 5.11 pg/ml, IQR: 0.87–19.9 pg/ml; Delta: median 6.1 pg/ml; IQR: 0.82–14.9) and in “no MS” subjects (ancestral: median 3.89 pg/ml, IQR: 1–79 pg/ml; Delta: median 4.56 pg/ml; IQR: 0.16–95.2; Figure 1A). A positive moderate correlation was found between the IFN-γ level in response to both ancestral and Delta spike (ancestral *r*_s_: 0.6, *P* < 0.0001; Delta *r*_s_: 0.5, *P* = 0.0004) and lymphocytes counts (Supplementary Figures 1C,D).

### IFN-γ-Specific Response Is Reduced in Patients Treated With Fingolimod or Cladribine

Subjects treated with fingolimod showed a significant lower IFN-γ-specific response to both the ancestral or the Delta pools compared to patients treated with ocrelizumab (*P* < 0.0001, both comparisons), cladribine (*P* = 0.0005, *P* = 0.0001, respectively) or IFN-β (*P* < 0.0001, both comparisons; Figure 1B, Table 2). Moreover, IFN-γ level in response to the ancestral pool was significantly lower in patients treated with cladribine compared to patients treated with ocrelizumab (*P* = 0.0024) or IFN-β-treated (*P* = 0.0042) (Table 2). The IFN-γ response to ancestral or Delta peptide pools was significantly reduced in patients treated with fingolimod compared to “no MS” subjects (*P* = 0.0039, *P* = 0.0041, respectively; Table 2). The evaluation of lymphocyte counts in patients with MS stratified based on the different therapies showed that patients taking fingolimod or cladribine had significant lower levels of lymphocytes compared to patients taking ocrelizumab or IFN-β (Supplementary Figure 1B).

**Table 2:** Medians and IQR of IFN-γ and anti-RBD IgG levels in MS patients and “no MS” subjects.

	**MS**				**No MS**
	**Ocrelizumab**	**Fingolimod**	**Cladribine**	**IFN-β**	
Ancestral spike IFN-γ Median pg/ml (IQR)	17.6 (11.8–23.9)	0 (0–0.1)	1.9 (0.7–3.5)	10.3 (5.1–25.1)	3.9 (1–79)
Delta spike IFN-γ Median pg/ml (IQR)	14.2 (9.7–23.3)	0 (0–0)	1.8 (1.1–23.9)	10.7 (5.6–17.7)	4.6 (0.2–95.2)
Anti-RBD IgG median BAU/ml (IQR)	0.4 (0.1–3.3)	3.4 (1.6–40.2)	84.2 (42.6–121.1)	126.4 (55.8–174)	100.4 (38.7–445.8)

*MS, multiple sclerosis; IQR, interquartile range; IFN, interferon; RBD, receptor binding domain; BAU, binding antibody unit*.

### Humoral Response Is Impaired in Patients Treated With Ocrelizumab or Fingolimod

All the enrolled subjects were anti-N IgG negative (data not shown). Patients with MS showed significant lower anti-RBD IgG levels (median 50.3 BAU/ml, IQR: 3–109.7) compared to “no MS” subjects (median 100.4 BAU/ml, IQR: 38.7–445.8; *P* = 0.0452; Figure 1C, Table 2). Patients treated with ocrelizumab or fingolimod had significant lower anti-RBD IgG levels compared to patients treated with cladribine (*P* < 0.0001, *P* = 0.0024, respectively) or IFN-β (*P* < 0.0001, *P* = 0.0004, respectively; Figure 1D, Table 2) and compared to “no MS” subjects (*P* < 0.0001, *P* = 0.0071, respectively; Table 2). All the “no MS” subjects scored positive to anti-RBD IgG (9/9, 100%), whereas, within the patients with MS 7/9 (77.8%) of patients treated with ocrelizumab and 5/9 (55.6%) or fingolimod were scored negative. Patients treated with cladribine or IFN-β scored all positive (Figure 1D). A significant low correlation was found between the anti-RBD IgG and the IFN-γ response the Delta spike (ancestral: *r*_s_ = 0.22, *P* = 0.1; Delta: *r*_s_ = 0.3, *P* = 0.02; data not shown).

## Discussion

SARS-CoV-2 vaccination schedules and vaccine-induced immune response are important issues in patients with MS. MS patients show variable vaccine-induced antibody response depending on the ongoing therapy (9, 19). Moreover, vaccine-induced cellular response, that is crucial for viral clearance and for a coordinated and successful immune response (5), may be impaired by DMTs (9). A robust T-cell response would be important to fill the protection gap in subjects with low/absent antibody response, also considering the emergence of new viral variants that may evade the antibody recognition.

**Figure 2:**
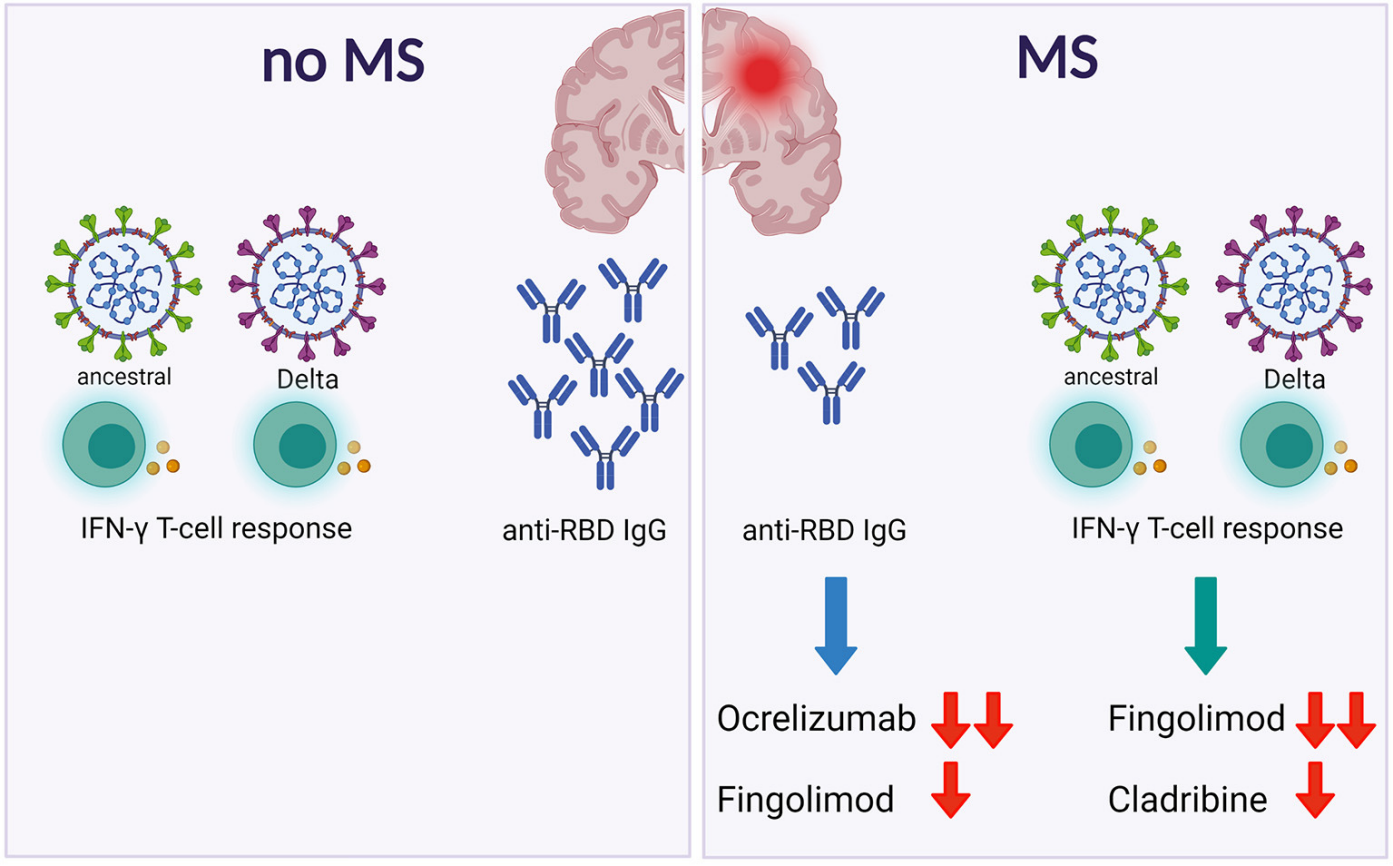
Patients with MS show a specific spike T-cell response to both ancestral or Delta SARSCoV-2. However, DMTs differently affect the antibody and the T-cell responses. Footnotes: MS, Multiple Sclerosis; IFN, Interferon; RBD, Receptor Binding Domain.

Here we showed that patients with MS mount a T-cell response to the Delta SARS-CoV-2 variant, although particular DMTs may be detrimental as previously shown for the ancestral strain (9) (Figure 2).

The analysis of the T-cell specific response showed that fingolimod and cladribine with a lesser extent, hampered the cellular response to either ancestral or Delta spike. These results are likely due to the selective mechanism of action of these drugs on T cells sequestration in the lymphoid tissues (fingolimod) or to the reduction of T and B cell proliferation (cladribine), with the result of the reduction of both immune populations. Likely, a third vaccine-dose may boost the T-cell memory response in these patients, as recently showed for MS patients on ocrelizumab (13). More importantly, the magnitude of T-cell response should be evaluated in terms of protection from new variants and from severe disease. Several methodologies have been used to measure natural or vaccine-induced T-cell responses, as intracellular cytokine staining, activation-induced markers and IFN-γ release based assay and each platform may have an impact on the generated results. Robust and standardized assays, easily implementable in the routine practice, are needed for T-cell measurements on a global scale (5) in order to evaluate potential correlates of protection.

Very recently, it has been demonstrated that vaccination by different COVID-19 vaccines in healthy donors induces a memory T-response that cross-recognize SARS-CoV-2 variants from Alpha to Omicron (20, 21). Therefore, although antibody titers against Omicron are low after vaccination schedule completion, and may be boosted after a third immunization, the T-cell response is largely intact. These data are encouraging as demonstrated that, despite the continuous viral variation, the T-cell responses will provide a second line of defense in case of immunological escape from antibodies (20). Likely, this cross-protection could be translated to MS patients, at least in those patients with a certain degree of T-cell response, and should be carefully evaluated in patients treated with DMTs acting on T cells.

The antibody response analysis showed an impaired antibody response in patients treated with ocrelizumab or fingolimod, whereas patients treated with cladribine or IFN-β showed a humoral response similar to controls. These findings confirm published data (9, 19) and highlight the importance of the evaluation of vaccine-induced protection in these subjects as measure to prevent persistent infections and severe disease. In particular, ocrelizumab impact on vaccine-induced immune response has been extensively evaluated. Several studies on SARS-CoV-2 humoral response demonstrated that anti-RBD IgG titers or serology positive rates were lower in vaccinated MS patients taking anti-CD20 compared to controls; however, T-cell responses were detectable 1–2 months after vaccination in the majority of the subjects evaluated (22–24). This study extends these concepts to a longer post-vaccination period.

Limitations of this study rely on the small number of the patients with MS evaluated and the lack of a calculation size for the study population. However, several DMTs were taken into consideration, allowing a more comprehensive understanding of the vaccine-induced immune response to viral variants in the real-life scenario of the MS disease. Moreover, within the “No MS” group, viral-vectors vaccinated subjects were included. However, this setting may reflect the true vaccination status of the “non-fragile” population in our country. Moreover, even excluding these subjects from the analysis, no significant differences were observed regarding the T-cell response in comparison to the MS group.

In conclusion, here we showed that patients with MS show a specific spike T-cell response to both ancestral or Delta SARS-CoV-2. However, DMTs differently affect the antibody and the T-cell responses (Figure 2). These findings, if confirmed in larger studies, may guide monitoring of patients with MS during vaccination.

## Data Availability Statement

The raw data generated and/or analyzed within the present study are available in our institutional repository (rawdata.inmi.it), subject to registration. The data can be found by selecting the article of interest from a list of articles ordered by year of publication. No charge for granting access to data is required. In the event of a malfunction of the application, the request can be sent directly by e-mail to biblioteca@inmi.it.

## Ethics Statement

The studies involving human participants were reviewed and approved by Ethical Committee of San Camillo Forlanini Hospital (Approval number 831/CE Lazio 1) and of National Institute for Infectious Diseases L. Spallanzani (Approval numbers 297/2021, 319/2021, 72/2015, and 59/2020). The patients/participants provided their written informed consent to participate in this study.

## Author Contributions

DG and EN wrote the projects to be submitted to the Ethical Committee. DG, EN, CT, and CG conceived and designed the study. Experiments were performed by AA, CF, SM, and VV. LPe performed the data analysis. CT, SR, FP, LPr, SH, GC, SG, CG, and EN enrolled patients and collected clinical data. LPe, CT, CC, AG, AS, and DG drafted the article or revised it critically. All authors contributed to the article and approved the submitted version.

## Funding

This project has been funded in whole or in part with Federal funds from the National Institute of Allergy and Infectious Diseases, National Institutes of Health, Department of Health and Human Services, under Contract Nos. 75N93021C00016 and 75N93019C00065 to AS; by the Italian Ministry of Health, by INMI Lazzaro Spallanzani Ricerca Finalizzata COVID-2020-12371675 and Ricerca Corrente on emerging infections; by generous liberal donations funding for COVID-19 research from Esselunga S.p.A, Camera di Commercio, Industria e Artigianato di Roma, Società Numero Blu Servizi S.p.A., Fineco Bank S.p.A, Associazione magistrati della Corte dei conti, and Società Mocerino Frutta Secca s.r.l (resolutions no. 395 of May 25th 2021, no. 254 of April 24th 2021, and no. 257 of April 14th 2021). The funders were not involved in the study design, collection, analysis, and interpretation of data, the writing of this article, or the decision to submit it for publication.

## Conflict of Interest

AS is a consultant for Gritstone Bio, Flow Pharma, Arcturus Therapeutics, ImmunoScape, CellCarta, Avalia, Moderna, Fortress, and Repertoire. La Jolla Institute has filed for patent protection for various aspects of T cell epitope and vaccine design work. CT received honoraria for speaking, travel grants and advisory board from Biogen, Merck-Serono, Bayer-Schering, Teva, Sanofy, Roche, Mylan, Almirall, and Novartis; CG received fees as speaker or advisory board from Merck, Bayer, Biogen, Novartis, Teva, Sanofy, Roche Almiral, and Mylan; SR has received honoraria from Biogen, Merck Serono, Novartis, and Teva for consulting services, speaking and/or travel support; LPr received consulting fees and/or speaker honoraria from Biogen, Celgene, Genzyme, Merck- Serono, Novartis, and Teva, travel grants from Biogen, Genzyme, Novartis, and Teva, research grants from the Italian MS Society (Associazione Italiana Sclerosi Multipla) and Genzyme; SH received travel funding and/or speaker honoraria from Biogen, Roche, Genzyme, Novartis, and CSL Behring; SG received honoraria for speaking and travel grants from Biogen, Sanofi-Aventis, Merck Serono, Bayer-Schering, Teva, Genzyme, Almirall, and Novartis; EN is member of the advisory board by Gilead, Lilly, and Roche and received fees for educational training by Gilead, Lilly, and Roche; DG is member of the advisory board by Biomerieux and Eli-Lilly, and received fees for educational training or consultancy by Amgen, Biogen, Cellgene, Diasorin, Janssen, Qiagen, and Quidel. The remaining author declares that the research was conducted in the absence of any commercial or financial relationships that could be construed as a potential conflict of interest.

## Publisher's Note

All claims expressed in this article are solely those of the authors and do not necessarily represent those of their affiliated organizations, or those of the publisher, the editors and the reviewers. Any product that may be evaluated in this article, or claim that may be made by its manufacturer, is not guaranteed or endorsed by the publisher.

## Supplementary Material

Supplementary Figure 1 Patients with MS show lymphocyte counts similar to “no MS” subjects, although the different DMTs differently affect the counts. **(A)** No significant differences were found comparing the lymphocyte counts between MS patients and “no MS” subjects. **(B)** Patients with MS treated with fingolimod or cladribine showed significant lower lymphocyte counts compared to subjects treated with ocrelizumab or IFN-β. **(C)** Lymphocyte counts significantly correlated with IFN-γ-specific response to the ancestral spike. **(D)** Lymphocyte counts significantly correlated with IFN-γ-specific response to the Delta spike. Horizontal lines represent medians. IFN-γ levels were measured by ELLA. Mann–Whitney test was used for pairwise comparisons. Spearman's Rank test was used for correlations. MS, multiple sclerosis; IFN, interferon.
