# Supplementary figures and images for: Humoral and Cellular Response to Spike of Delta SARS-CoV-2 Variant in Vaccinated Patients With Multiple Sclerosis
Source: Front Neurol. 2022 May 31;13:881988. doi: 10.3389/fneur.2022.881988 (PMC9194677; doi:10.3389/fneur.2022.881988)

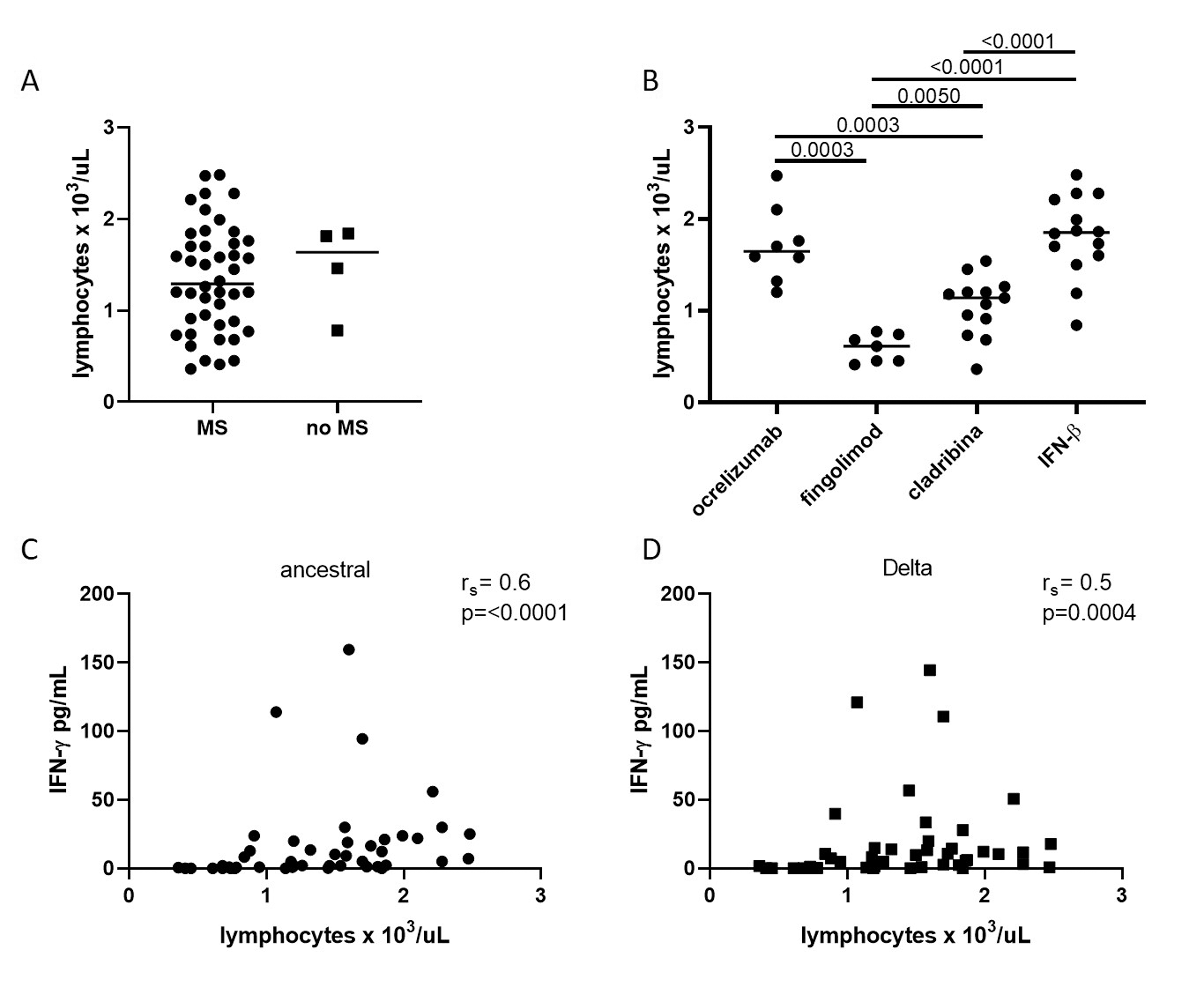

Supplement: Supplementary Figure 1 — Patients with MS show lymphocyte counts similar to “no MS” subjects, although the different DMTs differently affect the counts. (A) No significant differences were found comparing the lymphocyte counts between MS patients and “no MS” subjects. (B) Patients with MS treated with fingolimod or cladribine showed significant lower lymphocyte counts compared to subjects treated with ocrelizumab or IFN-β. (C) Lymphocyte counts significantly correlated with IFN-γ-specific response to the ancestral spike. (D) Lymphocyte counts significantly correlated with IFN-γ-specific response to the Delta spike. Horizontal lines represent medians. IFN-γ levels were measured by ELLA. Mann–Whitney test was used for pairwise comparisons. Spearman's Rank test was used for correlations. MS, multiple sclerosis; IFN, interferon. [file Image_1.TIF]
